# Osteochondral Lesions of the Subtalar Joint: Clinical Outcomes in 11 Patients

**DOI:** 10.1177/19476035231200339

**Published:** 2023-10-05

**Authors:** Tristan M.F. Buck, James J. Butler, Mohammad T. Azam, Carlijn ter Laak Bolk, Quinten G.H. Rikken, Matthew B. Weiss, Jari Dahmen, Sjoerd A.S. Stufkens, John G. Kennedy, Gino M.M.J. Kerkhoffs

**Affiliations:** 1 Department of Orthopedic Surgery and Sports Medicine, Amsterdam UMC, University of Amsterdam, Amsterdam, The Netherlands; 2 Amsterdam Movement Sciences, Musculoskeletal Health, Amsterdam, The Netherlands; 3 Academic Center for Evidence-Based Sports Medicine (ACES), Amsterdam UMC, Amsterdam, The Netherlands; 4 Amsterdam Collaboration on Health & Safety in Sports (ACHSS), IOC Research Center, Amsterdam UMC, Amsterdam, The Netherlands; 5 Department of Orthopedic Surgery, NYU Langone Health, New York, NY, USA

**Keywords:** osteochondral lesions, subtalar, clinical, radiological

## Abstract

**Objective:**

The purpose of this retrospective case series was to evaluate clinical outcomes following both conservative treatment and arthroscopic bone marrow stimulation (BMS) for the management of symptomatic subtalar osteochondral lesions (OCLs).

**Design:**

All symptomatic subtalar OCLs with a minimum of 12 months follow-up having undergone either a conservative management or arthroscopic procedure were included. Patient-reported outcomes were collected via questionnaires consisting of the Foot and Ankle Outcome Score (FAOS), Numeric Rating Scale (NRS) of pain in rest, during walking, during stair climbing, and during running. In addition, return to sports data, return to work data, reoperations, and complications were collected and assessed. In total, 11 patients across 2 academic institutions were included (3 males, 8 females). The median age was 43 years (interquartile range [IQR]: 32-53).

**Results:**

All patients underwent conservative treatment first; in addition, 9 patients underwent subtalar arthroscopic debridement with or without BMS. The median follow-up time was 15 months (IQR: 14-100). In the surgically treated group, the median NRS scores were 2 (IQR: 1-3) during rest, 3 (IQR: 2-4) during walking, 4 (IQR: 4-5) during stair climbing, 5 (IQR: 4-5) during running and the median FAOS score at final follow-up was 74 (IQR: 65-83). In the conservatively treated patients, the median NRS scores were all 0 (IQR: 0-0) and the median FAOS scores were 90 (IQR: 85-94). In the group of surgical treated patients, 4 were able to return to the same level of sports, 2 returned to a lower level of sports. Both conservatively treated patients returned to the sport and the same level of prior participation. All patients except one in the surgical group returned to work.

**Conclusions:**

This retrospective case series demonstrated that a high number of patients converted to surgery after initial conservative treatment. In addition, debridement and BMS show good clinical outcomes for the management of symptomatic subtalar OCLs at short-term follow-up. No complications nor secondary surgical procedures were noted in the surgically treated group. The high rate of failure of conservative treatment suggests that surgical intervention for symptomatic subtalar OCLs can be the primary treatment strategy; however, further research is warranted in light of the small number of patients.

## Introduction

An osteochondral lesion (OCL) refers to an injury to the articular cartilage and/or underlying subchondral bone and is frequently associated with a traumatic etiology.^1^ OCLs are most commonly encountered in the knee and ankle joint.^2,3^ In comparison, OCLs of the subtalar joint are rarely observed, thus the precise prevalence of this pathology is unknown. Subtalar OCLs can occur at either the articulating aspect of the talus or calcaneus of the subtalar joint following direct trauma to the joint secondary to a calcaneal fracture and/or acute ankle sprain.^4^ Patients can present with acute pain and swelling in or around the sinus tarsi exacerbated by ambulation. Symptomatic subtalar OCLs are often missed on initial evaluation due to the nonspecific nature of the presentation and poor sensitivity of plain film radiographs.^5^ Repeat imaging with computed tomography (CT) scans and/or magnetic resonance imaging (MRI) scans displays a focal lesion at the articular cartilage with potential surrounding bone marrow edema and underlying subchondral cysts.

Given the rare presentation of this debilitating pathology, no evidenced-based treatment paradigm currently exists. The literature is limited to a small number of case reports which vary in their approach to this pathology. Certain case reports describe successful treatment of patients with subtalar OCLs with conservative management which entails physical therapy and intra-articular corticosteroid and/or lidocaine injections.^6^ Conversely, further case reports demonstrated successful outcomes following surgical intervention via subtalar arthroscopy followed by surgical excision, curettage, and drilling of the OCL.^4^ Given the paucity of data that exists regarding clinical outcomes following treatment of subtalar OCLs, the optimal management approach remains unclear.

The purpose of this retrospective case series was to evaluate clinical outcomes following treatment of patients with both conservative management and surgical intervention for subtalar OCLs. Our hypothesis was that minimally invasive subtalar arthroscopic intervention would lead to improvement in subjective clinical outcomes together with good return to work and sporting activities at final follow-up with patients with subtalar OCLs.

## Methods

This multi-center retrospective case series used chart review for retrieval of data on individual patients following approval by the 2 local institutional review boards (Amsterdam UMC and NYU Langone Health). The study was performed in accordance with the Declaration of Helsinki. All patients who underwent treatment for OCLs of the subtalar joint by 3 attending foot and ankle surgeons as part of standard clinical care from January 5, 2000 to January 28, 2022 were identified. Inclusion and exclusion criteria are listed in **Table 1**.

**Table 1.** Inclusion and Exclusion Criteria.

Inclusion Criteria	Exclusion Criteria
Symptomatic osteochondral lesions to the subtalar joint having undergone a conservative treatment protocol or a localized surgical intervention (i.e., arthroscopic bone marrow stimulation/debridement) after failed previous conservative treatment.	Patients lost to follow-up or unwilling to participate.
Minimal follow-up of 12 months	End-stage osteoarthritis of the subtalar joint defined as joint destruction with an absent joint line on x-ray or CT.
	Concomitant disabling disease of the lower limb

CT = computed tomography.

### Data Collection

After initial screening of patient records, eligible patients were contacted by phone for participation in the study. In case of agreement and eligibility with participation the following baseline characteristics were collected: age, gender, follow-up time, and primary treatment strategy. In addition, questionnaires were sent with an electronic data capture system Castor^©^.

### Clinical Outcomes

Patient-reported outcome measurements that were collected at final follow-up include the Numeric Rating Scale (NRS) for pain during rest, walking, stair climbing and running, and the FAOS. Data regarding the return-to-work rate (not times) and the return to sport rate (not times) as well as complications, failures, and secondary surgical procedures were collected and assessed.

### Radiological Assessment

Baseline CT scans and/or MRI scans were used to measure the following radiologic outcomes: lesion location, morphology, coronal diameter, sagittal diameter, depth, surface of the lesion, and lesion volume. In case of radiological assessment with a CT and MRI, the CT scan was used for measurement of radiological parameters. Radiographic lesion characteristics were reported at each institution on 2 discreet occasions to establish intraobserver reliability. An independent from each institution performed the same assessment to establish interobserver reliability. A 2-way random intraclass correlation coefficient (ICC) model was used to calculate the interobserver agreement. The ICC was interpreted as poor (0.40), moderate (0.40-0.75), substantial (0.75-0.90), or excellent reliability (>0.90). All statistical analyses were performed with R statistical software (version 4.1.0, 2021; Foundation for Statistical Computing, Vienna, Austria).

### Data Analysis

All statistical analyses were performed with R statistical software (version 4.1.0, 2021; Foundation for Statistical Computing). Continuous variables were presented as median and interquartile ranges (IQR).

### Patient Demographics

In total, 11 patients (15 lesions) were included in this retrospective study. Patient demographic details are described in **Table 2**. Three patients were male, and 8 patients were female. The median age was 43 years (IQR: 32-53). The median body mass index (BMI) was 26 kg/m^2^.

**Table 2.** General Information.

Patient Number	Sex	Age	BMI	Follow Up Duration (Months)	Primary Treatment	Secondary Treatment
Conservative treatment						
1	F	46	25	170	Conservative	–
2	M	13	24	45	Conservative	–
Surgical treatment						
3	F	55	31	86	Conservative	BMS
4	M	38	27	114	Conservative	BMS
5	F	55	26	140	Conservative	BMS
6	F	49	28	14	Conservative	Debridement + PRP
7	F	59	26	12	Conservative	BMS
8	F	59	24	14	Conservative	BMS + CBMA
9	F	58	27	15	Conservative	BMS + CBMA
10	M	40	28	12	Conservative	BMS + CBMA
11	F	31	21	15	Conservative	Debridement
Average		43	26	15		
(median ± IQR)		(32-53)	(24-28)	(13.5-100)		

BMI = body mass index; M = male; F = female; R = right; L = left; BMS = bone marrow stimulation; PRP = platelet-rich plasma; CBMA = concentrated bone marrow aspirate; IQR = interquartile range.

#### Treatment techniques

An individualized approach was used for patients with an OCL of the subtalar joint to select the most appropriate treatment strategy. Influencing factors consist of personal factors and lesion characteristics. Personal factors considered in this process were age, level of activity, BMI, and patient preference. Lesion characteristics that played an important role in the choice of treatment consist of lesion size, lesion morphology, location, and prior treatment. Treatment strategies were most often conducted in an order from less invasive to more invasive as described below.

##### Conservative management

Conservative treatment was utilized in all recruited patients. This treatment strategy consists of several sub-strategies which can be combined with the main goal to enable patients’ full return to daily activities and desired level of sports. The first option for conservative management is treatment following the “supervised neglect” principle, in which patients adjusted their level of activity based on the severity of their complaints. Physical therapy was applied to increase strength and proprioception aiming for a higher functional level of the injured ankle. For appropriate distribution of forces across the subtalar joint, inlay soles were prescribed. Weight loss was advised to patients with overweight (BMI >25) to decrease the compressive load across the subtalar joint in an effort to reduce symptoms. Injection therapy consisted of hyaluronic acid (HA) injections and platelet-rich plasma (PRP) injections. The duration of therapy depends on patients’ individual goals, but if conservative management did not improve the level of symptoms as desired, or did not lead to the desired level of activities after 3 to 6 months, surgical interventions were considered.

#### Surgical technique: Arthroscopic bone marrow stimulation

##### Posterior ankle arthroscopy with bone marrow stimulation

**Figure 1.**
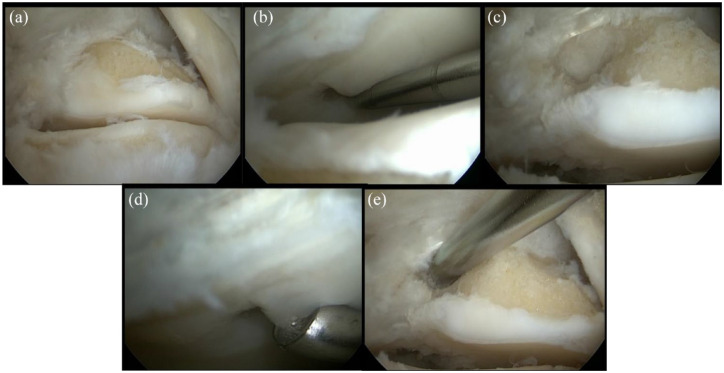
Intra-operative arthroscopic images of a left posterior ankle arthroscopy for the identification of the subtalar joint (**A**), identification and assessment of stability of a cystic osteochondral lesion of the subtalar joint on the talar side (**B** and **C**), removal of unhealthy and unstable borders of the osteochondral lesion with a curette (**D**), and removal of the cyst through the use of a curette (**E**).

All surgical procedures were performed with an arthroscopic approach. Before the procedure was started, patients received local anesthesia with a sciatic and saphenous nerve block performed by the anesthesia service. In addition, based on the patient’s preferences, additional general anesthesia was applied. Then, the patient was infused with a prophylactic 2 g cefazoline. The patient was placed in the prone position after which a sterile working field was created, and the operative field was scrubbed with chlorhexidine. Subsequently, a tourniquet was inflated around the upper leg to reach hemostasis. After these preparatory works, the incisions were made. The appropriate place for the posterolateral portal was identified by placing a probe on the distal tip on the lateral malleolus, parallel to the foot sole while the foot was kept in 90°. A line was drawn from the distal tip of the malleolus to the Achilles tendon after which the portal was made above this line 5 to 7 mm anterior to the Achilles tendon. The same approach can be used for the posteromedial portal with a line between the Achilles tendon and medial malleolus as marker. After creating the portals, the subtalar joint and flexor hallucis longus tendon were identified. If manual distraction was not sufficient, an extra soft tissue extractor was used. Then, (part of) the posterior tali process may removed to visualize the medial part of the subtalar joint. When only a debridement procedure had to be conducted, a shaver was introduced in the subtalar joint and the cyst was removed with this shaver. If an additional bone marrow stimulation (BMS) procedure was needed, a 4-mm chisel or microfracture awl was introduced to penetrate the subchondral bone (**Fig. 1** describes the step-by-step process).

If the OCL was located more laterally in the subtalar joint and could potentially not be visualized through a posterior ankle arthroscopy, a sinus tarsi arthroscopy was indicated. The sinus tarsi arthroscopy was conducted directly after the posterior ankle arthroscopy. For this procedure, an extra anterior portal was made in the subtalar joint. After creating this portal, a scope was introduced to visualize the needle which was introduced at the level of the posteromedial. An incision was made after the right identification of the needle to introduce the shaver. After introducing of the shaver, the anterior part of the subtalar joint was identified. If the right view was obtained, debridement was conducted with the shaver. An additional BMS procedure was optional to conduct by means of the introduction of a 4-mm chisel to penetrate the subchondral bone. After finishing the arthroscopy, both portals were closed with an Ethilon single suture and application of a circumferential compressing bandage for 2 to 3 days. Postoperative management consists of progression to full-weightbearing directly postoperatively with the usage of crutches for the first 2 to 6 weeks. A patient’s individualized and personalized tailored postoperative rehabilitation plan was designed together with an experienced foot and ankle specialized physiotherapist.

Following completion of the procedure, both portals were closed with an Ethilon single suture and application of a circumferential compressing bandage for 2 to 3 days. Postoperative management consisted of progression to full-weightbearing directly postoperatively with the usage of crutches for the first 2 to 6 weeks. A patient individualized and personalized tailored postoperative rehabilitation plan was designed together with a senior foot and ankle specialized physiotherapist.

## Results

### Radiological Assessment

**Figure 2.**
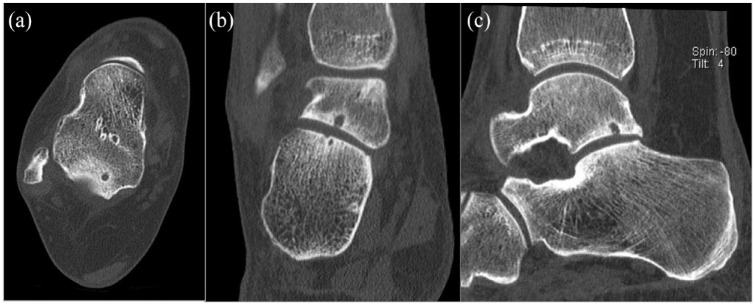
Talar-sided osteochondral lesion of the subtalar joint of a right ankle (note that there is also a small cystic lesion on the calcaneal side of the subtalar joint). (**A**) Axial view, (**B**) coronal view, and (**C**) sagittal view.

**Figure 3.**
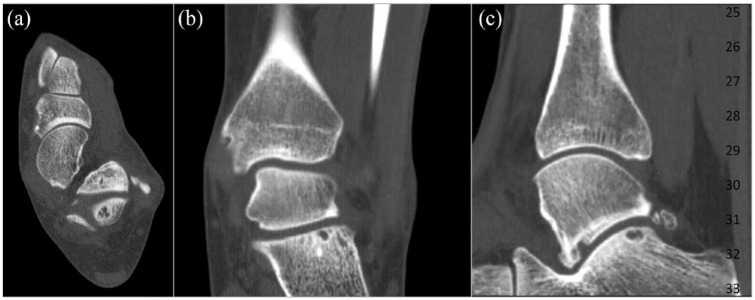
Calcaneal-sided osteochondral lesion of the subtalar joint of a left ankle. (**A**) Axial view, (**B**) coronal view, and (**C**) sagittal view.

The radiologic characteristics of the patients included in this study are described in **Table 3**. There were 3 right subtalar OCLs and 8 left subtalar OCLs. There were no cases of bilateral involvement. Four patients had a concomitant of a talar and calcaneal sided lesion and 4 patients only had involvement of the calcaneus. Three of the included patients had solely a talar-sided OCL. The calculated ICCs were all >0.90 (excellent). Pretreatment radiological examples of patients with subtalar OCLs are presented in **Figures 2** and **3**.

**Table 3.** Radiological Characteristics of the Included Patients.

Patient	Side	Location	Morphology	Sagittal Diameter (mm)	Coronal Diameter (mm)	Depth (mm)	Surface (cm^2^)	Volume (cm^3^)
Conservative treatment								
1	L	Calcaneus	Crater	5.3	6.2	2.8	0.26	0.05
2	R	Talus	Cystic	8.4	5.2	4.5	0.34	0.08
Surgical treatment								
3	L	Calcaneus	Cystic	5.5	3.2	4.5	0.14	0.04
	L	Talus	Cystic	2.3	3.5	7	0.06	0.02
4	L	Talus	Fragmentous	4.6	5.5	6	0.20	0.06
5	R	Calcaneus	Cystic	1.7	2.0	2.1	0.03	0.004
	R	Talus	Cystic	4.9	3.3	3.0	0.13	0.02
6	L	Talus	Cystic	NA	NA	NA	NA	NA
7	L	Calcaneus	Cystic	NA	NA	NA	NA	NA
8	L	Calcaneus	Cystic	10	6	2	0.47	0.06
	L	Talus	Cystic	1.6	1.3	0.9	0.01	0.001
9	R	Calcaneus	Cystic	5	4	1.5	0.16	0.02
10	L	Calcaneus	Cystic	1.9	3.7	1.3	0.25	0.03
	L	Talus	Cystic	6.5	4.8	1.6	0.08	0.01
11	L	Calcaneus	Cystic	10	6	2.1	0.74	0.07
Average				T: 4.1 mm C: 6.3 mm	T: 3.8 mm C: 4.6 mm	T: 3.8 mm C: 2.4 mm	T: 0.14 cm^2^ C: 0.25 cm^2^	T: 0.03 cm^2^ C: 0.04 cm^3^

T = talar; C = calcaneal; NA = not available; L = left; R = right.

### Clinical Outcomes

All clinical outcomes, including median and IQRs, are presented in **Table 4**. Nine of the 11 patients (82%) failed conservative management and subsequently underwent surgical intervention. Overall, the median FAOS score was 80 (IQR: 69-85), the median NRS at rest was 2 (IQR: 0-2), the median NRS when walking was 3 (IQR: 1-3), the median NRS when stair climbing was 4 (IQR: 1-5), and the median NRS when running was 3 (IQR: 2-5). The median follow-up time was 15 months (IQR: 13.5-100).

**Table 4.** Clinical Outcomes.

Patient Number	FAOS	NRS			
		Rest	Walking	Stair Climbing	Running
Conservative treatment					
1	81	0	0	0	0
2	98	0	0	0	0
Surgical treatment					
3	65	0	2	2	5
4	90	0	0	0	0
5	47	5	7	8	10
6	64	3	5	5	5
7	83	2	2	4	4
8	74	2	4	4	5
9	86	1	3	4	4
10	80	2	3	5	4
11	72	3	3	5	5
Average: median (IQR)	80 (69-85)	2 (0-3)	3 (1-3)	4 (1-5)	4 (2-5)

FAOS = Foot and Ankle Outcome Score; NRS = Numeric Rating Scale; IQR = interquartile range.

### Return to Sport and Work

Data concerning return to sport are described in **Table 5**. Eleven patients performed one or more type of sports prior to the injury and 2 patients did not perform any type of sports before the injury. In the conservatively treated group, both patients returned to the same level of sports. In the surgically treated group, 6 patients (75%) returned to sport, with 4 patients (50%) returning to the same level of prior sporting activity.

**Table 5.** Return to Sport Outcomes.

Patient Number	Type of Sports Before Injury	Level of Sports Before Injury	Type of Sports After Treatment	Level of Sports After Treatment
Conservative treatment				
1	Running	Recreational	Running	Recreational
2	Walking, gymnastics	Recreational	Walking, gymnastics	Recreational
Surgical treatment				
3	Volleyball	Competitive	No sports	No sports
4	Ballet dancer	Professional	Crossfit	Recreational
5	Fitness	Recreational	No sports	No sports
6	Walking, Pilates	Recreational	Walking, Pilates	Recreational
7	Walking, weightlifting, dancing-aerobics	Recreational	Walking, weightlifting, dancing-aerobics	Recreational
8	Walking, swimming	Recreational	Walking, swimming	Recreational
9	NA	NA	NA	NA
10	Biking, running, tennis, soccer, squash	Competitive	Biking, running, tennis, soccer, squash	Recreational
11	Basketball, running	Recreational	Basketball, running	Recreational

NA = not available.

Data concerning return to work are described in **Table 6**. Ten patients (90.1%) returned to work. The 1 patient who did not return to work was a professional athlete and had to change sports due to ankle complains.

**Table 6.** Return to Work Outcomes.

Patient Number	Type of Work	Return to Work
Conservative treatment		
1	Office employee	Yes
2	Student	Yes
Surgical treatment		
3	Student	Yes
4	Professional athlete	No
5	Account manager	Yes
6	Teacher	NA
7	Social worker	Yes
8	Nurse	Yes
9	Nurse	Yes
10	Trader	Yes
11	Supervision investigator	Yes

NA = not available.

### Complications and Reoperations

No complications nor secondary surgical procedures were observed.

## Discussion

The most important finding from this retrospective case series was that surgical intervention for the management of symptomatic subtalar OCLs led to good clinical outcomes at short-term follow-up. In addition, no complications nor secondary surgical procedures were observed in this study. The high rate of failure of conservative treatment in this patient population suggests that early surgical intervention for symptomatic subtalar OCLs following a short period of conservative management may be the primary treatment strategy; however, further research is warranted.

Symptomatic OCLs of the subtalar joint are a rarely encountered pathology with the vast majority of the literature surrounding symptomatic subtalar OCLs describing only case reports.^4^ These lesions often go undetected due to the nonspecific nature of the patient’s symptomatology and poor sensitivity of plain film radiographs.^4^ In light of the paucity of literature that exists for subtalar OCLs, no evidence-based treatment guidelines have been described to date. Small, asymptomatic subtalar OCLs detected incidentally may be treated conservatively with a “watch and wait” approach. However, symptomatic subtalar OCLs will require intervention in the form of either conservative management entailing physical therapy or intra-articular injections of HA or PRP. If conservative management fails, surgical treatment is warranted. All 11 patients in this retrospective case series were initially trialed with conservative management which entailed activity modification, oral analgesics, weight loss, and intra-articular injection of PRP and/or HA. Nine of the 11 patients (82%) did not respond to conservative management and thus underwent surgical intervention via subtalar arthroscopic debridement and/or BMS. This suggests that early surgical intervention following a brief trial period of conservative treatment may be the primary treatment strategy for patients with symptomatic subtalar OCLs. However, given the small number of included patients, it is difficult to ascertain the optimal initial treatment approach; thus, further research warranted.

Few studies exist in the literature evaluating outcomes following surgical management of subtalar OCLs. A retrospective case series by Choi and Ogilvie-Harris^7^ examined outcomes in patients treated with both conservative management and surgical treatment in 10 patients with occult subtalar osteochondral fractures. Seven patients were treated surgically indicated by severe restriction of subtalar motion together with degenerative changes on radiographic imaging, which included 6 cases of subtalar arthrodesis and 1 case of triple arthrodesis, with 6 of the 7 patients presenting as asymptomatic at final follow-up. The 3 patients who underwent conservative management remained symptomatic at final follow-up. Furthermore, a case report by Kadakia and Sarkar^4^ described the management of a 14-year-old patient presenting with a symptomatic subtalar OCL. She failed initial conservative management and subsequently underwent subtalar arthroscopy followed by surgical excision, curettage, and drilling through a mini-arthrotomy approach. At 1-year follow-up, the patient was asymptomatic and resumed prior sporting activities.

Despite the limited available evidence, it is our recommendation that small- to moderate-sized symptomatic subtalar OCLs that fail conservative management can be managed with minimally invasive surgical techniques such as subtalar arthroscopic debridement and BMS. Subtalar arthroscopy allows for direct visualization of the subtalar articular joint surfaces with concomitant treatment of any identified intra-articular pathology.^8^ This arthroscopic approach avoids extensive joint dissection and disruption of the soft tissue envelope, thereby reducing the risk of postoperative wound complications while facilitating accelerated return to activities compared to traditional open techniques.^9^ This is reflected in this current retrospective study as no wound complications nor infections were observed in this study. In addition, arthroscopic evaluation of OCLs has been demonstrated to provide superior assessment of the size of OCLs in comparison with MRI,^1^ thus making subtalar arthroscopic evaluation an attractive diagnostic modality. BMS involves resection of unstable cartilage and underlying necrotic bone in conjunction with perforating the subchondral plate with an awl or pick to establish a communication with the subchondral bone marrow compartment to facilitate the migration of mesenchymal stem cells and growth factors, in an effort to promote the formation of reparative cartilaginous tissue.^10,11^ BMS with standard size picks for OCLs is associated with inferior clinical outcomes due to the de-differentiation of the initial type II collagen to type I collagen together with the irreparable damage sustained by the underlying subchondral bone.^12,13^ In an effort to mitigate against the destruction of the micro-architecture of the subchondral bone, we recommend the use of smaller, 1-mm picks when performing BMS. A study by Gianakos et al.^14^ found less talar dome trabecular compaction and sclerosis assessed via micro-CT analysis in specimens that underwent BMS with a 1-mm pick compared with 2-mm picks. In our study, the median postoperative FAOS and visual analog scale (VAS) scores were 80 and 2, respectively, suggesting good subjective clinical outcomes following arthroscopic BMS.

This current retrospective review evaluated return to sporting activity data in our study. In total, 75% of the patients who underwent surgical intervention for subtalar OCL returned to sporting activity at a mean return to play time of x. In addition, only 66.7% of the patients returned to their prior level of activity. These results are inferior to return to play data reported following BMS for the treatment of OLT.^15^ A systematic review of 3072 patients by Hurley et al. demonstrated an 86.8% return to play rate at a mean return to play time of 4.5 months following BMS for the management of OLT.^16^ It is difficult to ascertain the precise reason for the lower levels of return to play observed in our study, primarily due to the small sample size and heterogeneity between the surgical procedures carried out across the 2 institutions by 2 surgeons. Nevertheless, to optimize return to play in our study, we recommend following the guidelines described by at the 2017 International Consensus Meeting on Cartilage Repair of the Ankle.^17^ We encourage early range of motion exercises together with early progressive weightbearing to stimulate the formation of reparative cartilaginous tissue and to reduce the risk of sarcopenia of the surrounding supporting musculature and the formation of excessive scar tissue.

OCLs are difficult to treat pathologies in light of the poor regenerative capacity of the articular cartilage. As a result, novel biological adjuncts have been developed to augment the reparative process. cBMA is a biological adjuvant that is harvested from the ipsilateral iliac crest and is subsequently centrifuged to yield a product with a high concentration of growth factors—interleukin-1 receptor antagonist protein and mesenchymal stem cells.^18,19^ Concentrated Bone Marrow Aspirate (cBMA) has been studied extensively in the setting of OCLs of the talus. A retrospective case-control study by Hannon et al.^19^ compared outcomes between patients who underwent BMS with cBMA and patients who underwent BMS alone for the treatment of OLT. The authors demonstrated comparable postoperative FAOS and 12-item Short Form Survey (SF-12) scores between the 2 cohorts, but superior MOCART (Magnetic Resonance Observation of Cartilage Repair Tissue) scores and T2-mapping values in the BMS with cBMA cohort compared to the BMS-alone cohort, indicating that cBMA promotes improved border repair tissue integration following BMS. Three patients in this current retrospective study underwent BMS augmented with cBMA. All patients reported good postoperative FAOS scores and returned to their previous level of sport. The precise role of orthobiologics in the setting of subtalar OCLs remains uncertain; however, the chondroprotective effect of cBMA establishes it as a promising adjunct to improve the quality of the reparative cartilage.

### Limitations

There numerous limitations with respect to this study. The primary limitation is the small number of patients together with the non-comparative retrospective nature of this review. The short-term follow-up limits the understanding of the longevity of the BMS procedure for subtalar OCLs. Finally, there was variation in both lesion size and the implementation of surgical techniques across the 11 patients introducing significant heterogeneity into the study.

## Conclusion

This retrospective case series demonstrated that surgical intervention for the management of symptomatic subtalar OCLs led to good clinical outcomes at short-term follow-up. In addition, no complications nor secondary surgical procedures were in encountered in this study. The high rate of failure of conservative treatment in this patient population suggests that surgical intervention for symptomatic subtalar OCLs may be the primary treatment strategy; however, further research is warranted in light of the small number of patients.
